# Latticed pentamode acoustic cloak

**DOI:** 10.1038/srep15745

**Published:** 2015-10-27

**Authors:** Yi Chen, Xiaoning Liu, Gengkai Hu

**Affiliations:** 1 Key Laboratory of Dynamics and Control of Flight Vehicle, Ministry of Education, School of Aerospace Engineering, Beijing Institute of Technology, Beijing 100081, China

## Abstract

We report in this work a practical design of pentamode acoustic cloak with microstructure. The proposed cloak is assembled by pentamode lattice made of a single-phase solid material. The function of rerouting acoustic wave round an obstacle has been demonstrated numerically. It is also revealed that shear related resonance due to weak shear resistance in practical pentamode lattices punctures broadband feature predicted based on ideal pentamode cloak. As a consequence, the latticed pentamode cloak can only conceal the obstacle in segmented frequency ranges. We have also shown that the shear resonance can be largely reduced by introducing material damping, and an improved broadband performance can be achieved. These works pave the way for experimental demonstration of pentamode acoustic cloak.

Cloaking a target from detective wave is a fascinating topic attracting many researchers. Material design for such cloak is only possible recently based on transformation method, initially advanced for electromagnetic waves^1^,^2^ and extended subsequently to other waves^3^,^4^,^5^,^6^,^7^,^8^. Acoustic cloak was first explored by recognizing similarity between acoustic and electromagnetic wave equations^4^,^5^. Resulting acoustic cloaks have to resort to metafluids with isotropic stiffness but anisotropic density, which can be realized effectively by metamaterial technology^9^,^10^,^11^. Acoustic cloaks with exotic inertias are classified as inertial cloak (IC) after Norris^12^. Along this line, a number of works were carried out and experimental demonstrations were also evidenced^13^,^14^,^15^.

An alternative route leading to acoustic cloak is to make use of *pentamode* (PM) material. PM materials, after Milton and Cherkaev^16^, are a type of degenerated elastic materials characterized by elastic tensor 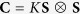, with 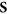 being a 2^nd^ order symmetric tensor. So *K* is the only non-zero eigenvalue of the elastic matrix, and a PM material can support only one stress state proportional to 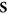, i.e. 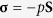, where the scalar *p* is named as pseudo pressure. Norris^12^ proved that under a space mapping, transformed acoustic wave equation with PM material expressed in pseudo pressure is form invariant. With such extension of transformation acoustics, the resulting cloak invokes PM materials with both anisotropic stiffness and anisotropic density, covering the IC as a special case. PM cloak discussed in this work refers to another special case where cloak material is pure PM^17^, i.e., with anisotropic stiffness but isotropic density. PM cloaks have several advantages over classical IC: they avoid mass singularity; they can be engineered with pure solid materials; they invoke only quasistatic stiffness of PM material and hence are theoretically broadband. It should be noted that though PM cloaks are free of mass singularity, they possess instead stiffness singularity. It is relatively easier to design materials with a wide range of stiffness than that of density.

Since then much progress has been made for PM cloaks. Gokhale *et al.*^18^ discussed choice of transformation function to get desirable material distribution. Scandrett *et al.*^19^ studied layered approximation of a spherical PM cloak. Fueled by transformation acoustics based on PM materials, investigation on PM materials themselves becomes also active recently. Kadic *et al.*^20^,^21^ explored property of a three dimensional (3D) PM lattice, and more recently Bückmann *et al.*^22^ applied this kind of PM lattice in a cloak shell to shield static force. Layman *et al.*^23^ proposed a two dimensional (2D) PM lattice and numerically verified its property with an acoustic mirage device. For PM acoustic cloak, though it has theoretically been proven to be possible for years, it is unclear whether it stands with practically available PM materials. In this article, we report a practical design of PM acoustic cloak assembled by graded PM lattices engineered with a single type of solid material. Effect of weak shear resistance inherent in PM lattices on cloaking performance is also demonstrated. These works pave the way for its experimental demonstration.

## Results

### PM material in cylindrical acoustic cloak

**Figure 1:**
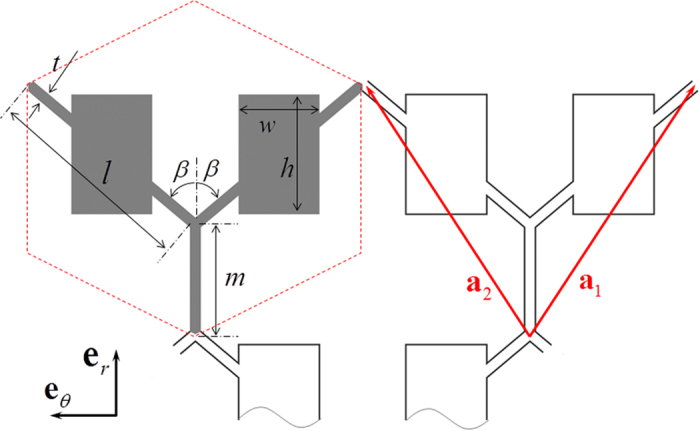
Microstructure of 2D PM lattice, with a highlighted unit cell. The cell geometry is characterized by length *m* of the vertical strut, length *l* of the other two struts, strut thickness *t*, angle *β*, and the size of the rectangular weights (*w*, *h*). The lattice possesses orthotropy in local **e**_*r*_ − **e**_*θ*_ frame, and the angle *β* plays an essential role on tuning wave speed anisotropy.

A cylindrical cloak with coordinate origin as its center calls for 2D radially symmetric transformation defined by 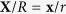, where 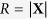, 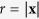, and 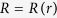 is mapping function. PM material in cloak shell is characterized by density 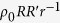 and elastic tensor 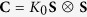, where 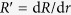, 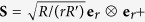 
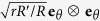
 with 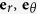 being base vectors of a polar system (c.f. Fig. 1), and 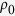 and 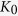 density and bulk modulus of background fluid, respectively^12^. It is more convienent to express material properties of the cloak in matrix form as:


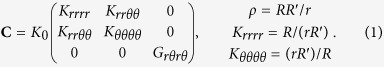


For a perfect PM material, conditions 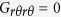 and 
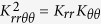
 should be satisfied in order to ensure unique non-zero eigenvalue of the elastic matrix. In particular, 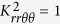 for present cylindrical cloak. However, a practical PM material made of solids is inherently imperfect since small shear resistance is not only unavoidable, but also necessary for stability of structure^21^,^22^,^23^. Therefore we can only expect for a practical PM material


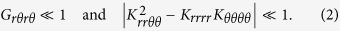


To avoid singularity of material parameters, we consider a transformation which maps an annular virtual domain 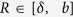 to physical one 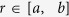, with 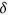 < *a*. To facilitate following microstructure implementation, we adopt mapping function 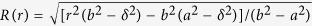, which leads to constant density along radial direction^18^.

### PM microstructure and construction of latticed cloak

The proposed PM lattice, as well as six parameters defining unit cell, are depicted in Fig. 1. The lattice can be considered as a honeycomb with two rectangular weights attached onto its two legs. As long as propagating wave length is much longer than lattice constant, quasistatic effective property is size-independent. To facilitate construction of graded cloak structure, the lattice is tuned by five dimensionless parameters 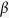, 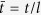, 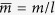, 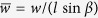 and 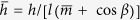, while absolute size of the unit cell is irrelevant. The base material of the lattice is chosen as Aluminum (density 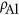 = 2700 kg/m^3^, bulk modulus 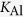 = 67.65 GPa, shear modulus 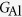 = 25.94 GPa), and the background fluid is water (ρ_0_ = 1000 kg/m^3^, *K*_0_ = 2.25 GPa). The proposed PM lattice is not perfect, and actually it is homogenized as an in-plane orthotropic elastic material with property given by equation (1) in principal axial system.

Since there is no resonance, we simply calculate effective density 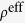 by volume average (more details can be found in Supplemental material). The effective elastic constants can be estimated from phase velocities 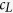 and 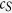 of P- and S- waves along principal directions, respectively, and velocities 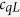 and 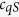 of quasi-P and quasi-S waves along another direction:


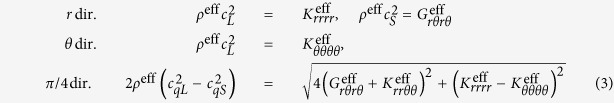


The phase velocities at longwave limit are obtained by Bloch wave analysis on one unit cell according to lattice vectors 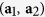 shown in Fig. 1. Task on material level is to find microstructure whose homogenized property matches the functional property (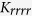, 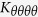, 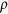) given by equation (1). In addition, equation (2) should at the same time be guaranteed to ensure proper PM behavior. It is noted that most existing studies check only equation (2)_1_ in design of PM materials and ignore equation (2)_2_. However, our calculation reveals that violation of any condition will damage performance of PM cloak. Mapping between the required property of the cloak and the corresponding microstructural parameters can be established by a minimization procedure:


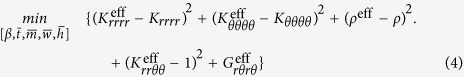


**Figure 2:**
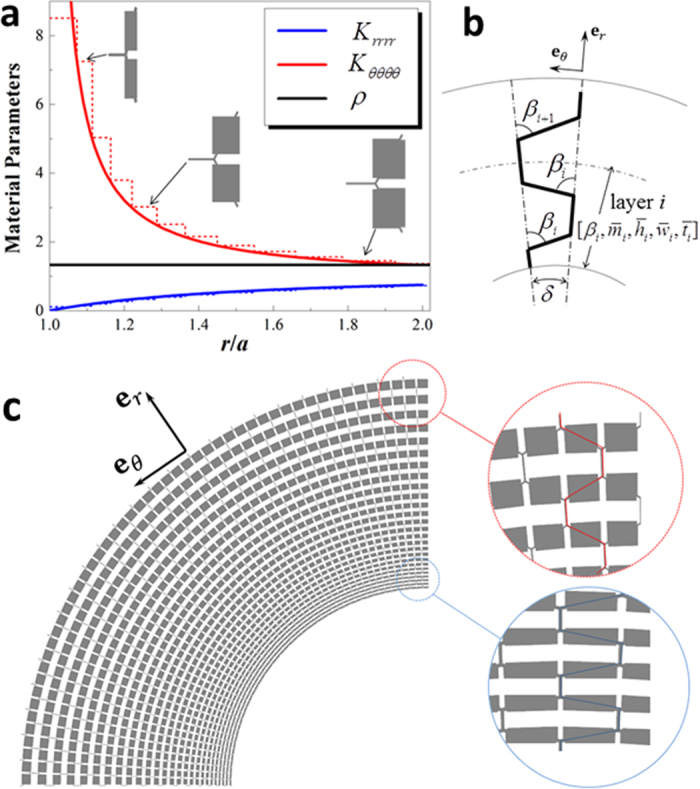
Latticed PM acoustic cloak design. (**a**) Profiles of continuously varying (solid lines) material property of the cloak shell, and their layered approximation (dashed lines) by the PM lattice, with cell geometry shown for the layers 2, 5 and 11. (**b**) Schematic illustration of the recursive implantation of lattice cells into cylindrical layers. (**c**) Layout of the latticed PM cloak, with the layer 1 being discretized by 4 cells, and the others by 2 cells each (totally 26).

Implementation of latticed PM cloak proceeds with layered approximation. Figure 2a shows continuous profile of material property determined by equation (1) and its layered approximation, where 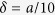 and 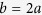 are used. Figure 2b sketches strategy of implanting lattice cells of local rectangular frame into cylindrical layer in a single sector (the sector angle 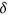 depends on circumferential discretization). Centerlines of the PM lattice struts are recursively determined by starting from the inner side and maintaining the parameters 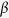 and 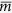 fixed layer by layer, and the PM lattice can then be developed out by using (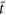, 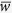, 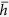). Figure 2c shows layout of a quarter of the designed latticed PM cloak, for which detailed microstructural parameters and homogenized effective stiffness for each layer are listed in Table 1. The cloak is divided into 100 sectors (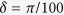) in 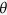 direction, and totally 26 cells are used to implement 12 homogeneous layers illustrated in Fig. 2a. Notice that the larger 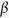 in the inner side of the cloak the more difference between wave speeds in 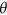-direction and *r*-direction, this anisotropy in wave speed is essential for redirecting wave around an obstacle. Except that the 1^st^ layer is approximated by the most extreme anisotropy achievable by the current lattice (
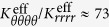
), effective stiffness of all the other layers precisely match the desired 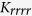 and 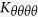. On the other hand, errors for 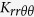 and 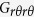 are limited below 6% and 4% compared to their ideal values, 1.0 and 0.0, respectively.

### Evaluation of cloaking performance

In this section, we will evaluate performance of the proposed cloak on shielding a sound-hard obstacle under plane acoustic wave. Full wave simulation was carried out by acoustic-structure module of finite element solver COMSOL Multiphysics, which solves elastodynamic equation in the latticed cloak and acoustic equation in the background water, respectively. Total scattering cross section (TSCS), defined as ratio of scattered power in all directions to incident power of plane wave, is employed to quantify wave scattering. For comparison and better understanding, analytical solutions for scattering of layered cylindrical cloak with perfect and imperfect PM materials are also developed. The details can be found in the Supplementary Information.

**Figure 3:**
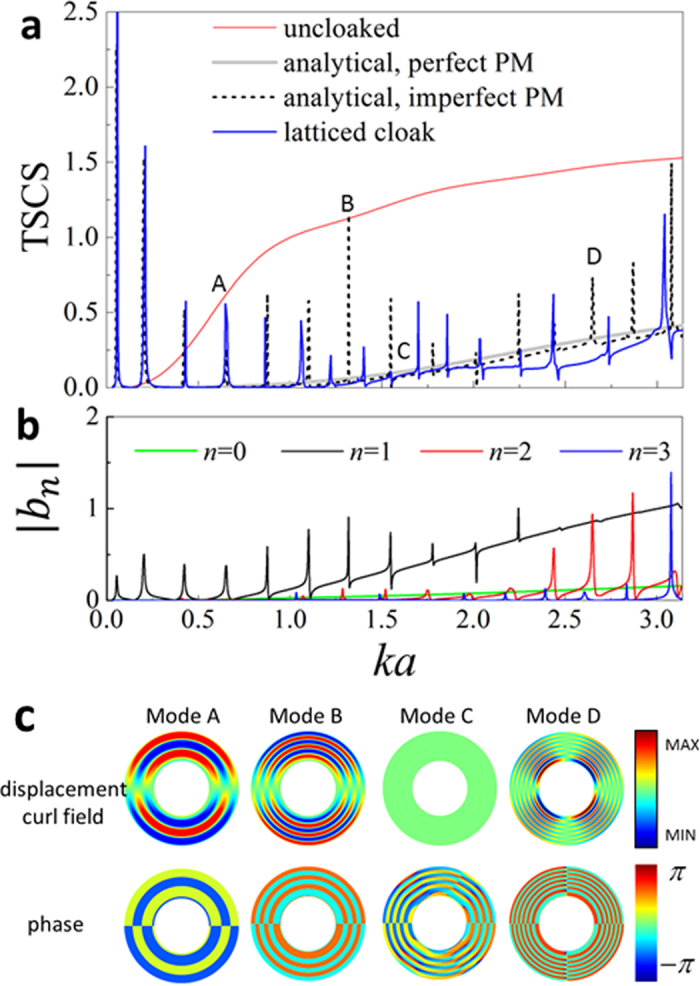
TSCS spectra of PM acoustic cloak. (**a**) Comparison of TSCS spectra for lattice cloak, layered cloaks with perfect and imperfect PM materials and uncloaked case. (**b**) Scattering coefficient of order 0 ~ 3 for the layered cloak with imperfect PM material. (**c**) Displacement curl fields and phase diagram corresponding to three resonance modes (**A,B,D**) and one non-resonance mode C as indicated in (**a**).

An obvious expectation upon the PM cloak is its broadband capability since it needs merely quasistatic property of the PM material and no resonant mechanism is invoked. The TSCS of the latticed cloak as well as the homogenized layered cloak are plotted in Fig. 3a as function of incident wave number 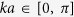. At 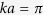, the wave length is about 25 times of the maximum cell size in the latticed cloak, thus the homogenization condition is satisfied in considered frequency range. For the layered cloak with the imperfect PM material, the thickness and homogenized property of each layer are taken from Table 1, and the same for the layered cloak with the perfect PM material except that ideal values 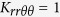 and 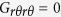 are used. It is seen from the figure that over most region of investigated frequency range the designed cloak significantly reduces the scattering and at least 25% reduction in TSCS is observed, compared to the uncloaked case. Overall, TSCS of the three cloaking cases increase with frequency due to the layered approximation of continuously varying material parameters, which is also a characteristic of layered IC^24^,^25^. Unfortunately, for the latticed cloak and the homogenized layered cloak with the imperfect PM material, the broadband nature is damaged by occurrence of quite a number of narrow resonance peaks. The TSCS curve of the latticed cloak, including the resonance peaks, matches very well with that of its homogenized counterpart (analytical, imperfect PM) under long wave length approximation (*ka* < 1), and discrepancy observed in higher frequency should be attributed to lattice discretization. Agreement of the analytical and numerical TSCS is an encouraging result that confirms the current homogenization and design process.

The resonance peaks in TSCS are remarkable and need more discussion. When concealing penetrable obstacles, the TSCS spectra of layered IC often displays peaks due to cavity resonance which has been well discussed in the literature^25^. Obviously, here the peaks are not of this type since the obstacle is rigid, but should be attributed to weak shear resistance of the imperfect PM material. For the case of the perfect PM material, wave energy transmitted into the cloak shell can only be supported by a single type of wave governed by scalar pseudo pressure equation, and is well guided by proper distribution of PM materials in the cloak layer. For the case of the imperfect PM material, besides the pseudo pressure wave, shear related wave modes corresponding to two easy-modes^16^ of a 2D PM material can also be excited inside the cloak. Since the PM lattice are designed to ensure 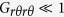 and 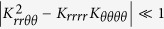, shear wave modes other than the pseudo pressure are not pronounced and do not damage much concealing effect in most region of the spectra. However, the shear modes are out of control since the PM cloak is designed to work only for (pseudo) pressure wave, and hence can form resonance at some frequencies leading to strong scattering even higher than the uncloaked case. To evidence this, for the layered cloak with the imperfect PM material, we examine in Fig. 3c three resonance modes at *ka* = 0.65 (Mode A), *ka* = 1.32 (Mode B), *ka* = 2.65 (Mode D) , and one mode *ka* = 1.63 (Mode C) at flat region, as marked in Fig. 3a. The displacement curl fields inside of the cloak shell are shown to check out shear related fields, and the corresponding field phase diagrams are also accompanied. For the modes A, B and D, very intense displacement curl field patterns and alternately phase reversal by 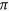 are presented, indicating clearly occurrence of resonance. Conversely for the non-resonant mode C, the shear field is negligible and the field phase is progressive different from a standing wave. Further, we also shown in Fig. 3b the first 4 orders of the scattering coefficients |*b*_*n*_| (*n* = 0 ~ 3) calculated by the analytical formulation. It is seen that in the considered frequency region, the resonance peaks are covered by the scattering coefficients of order 1 ~ 3 and one-by-one correspondence can be obviously recognized. It should be emphasized that the zero order coefficient *b*_0_ is consistently suppressed by the cloak and does not contribute to the resonance peaks, which is another proof that the resonance is shear related, since the resonance coupled to *b*_0_ can only be of shear-irrelavent monopole type. From the scattering coefficient spectra, the resonance Modes A and B are associated to *b*_1_ and should be of dipole type, while the Mode D is associated to *b*_2_ and is of quadrupole type, as seen in Fig. 3c. By observing structure of the |*b*_*n*_| spectra, we can anticipate that the resonance peaks will theoretically extend to full *ka* range, and segmentally be dominated by a certain order of *b*_*n*_ coupling to particular resonance type.

We have also analytically investigated the TSCS of layered cloaks with different degree of PM material imperfection. It is found that when errors in 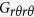 and 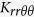 decrease, the resonance peaks tend to be more narrow and the spaces between the peaks shrink, hence the peaks aggregate towards low frequency end and till vanish. As it is impossible to have a perfect PM material in practice, the possibility of tuning 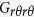 and 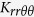 to control peak distribution at will or even to suppress them, remains an open problem and deserves future investigation. It is also interesting that the TSCS curves of the latticed cloak and the layered cloak with the imperfect PM material are below that of the layered cloak with the perfect PM material. By observing the spectra in Fig. 3a,b, we can find that a resonance peak always accompanied by a resonance dip, physically it implies that a scattering enhancement is always neighbored by a scattering cancellation. When the resonance peaks are not distant to each other, it transpires that the dips connect to make the TSCS curve with resonances lower than that without resonances in the region outside the peaks. Similar gains from resonance are also found for the non-perfect IC with small shear modulus^26^ and for the IC with penetrable obstacle^24^,^25^.

**Figure 4:**
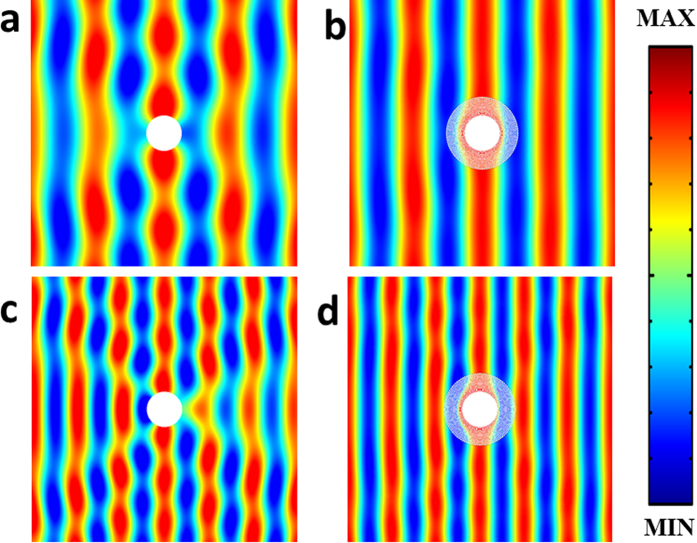
Scattering pressure field snapshots. (**a**) uncloaked, *ka* = 1.57. (**b**) Latticed cloak, *ka* = 1.57. (**c**) Uncloaked, *ka* = 2.51. (**d**) Latticed cloak, *ka* = 2.51.

In Fig. 4, we report two snapshots of scattering acoustic pressure field for the latticed cloak (right panels) at two frequencies. Inside the solid materials of the latticed cloak shell, hydrostatic stress field is plotted. Compared to the uncloaked cases (left panels), we get TSCS reduction 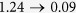 and 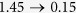 for *ka* = 1.57 and *ka* = 2.51, respectively, and a significant reduction in scattering is found.

## Discussions

**Figure 5:**
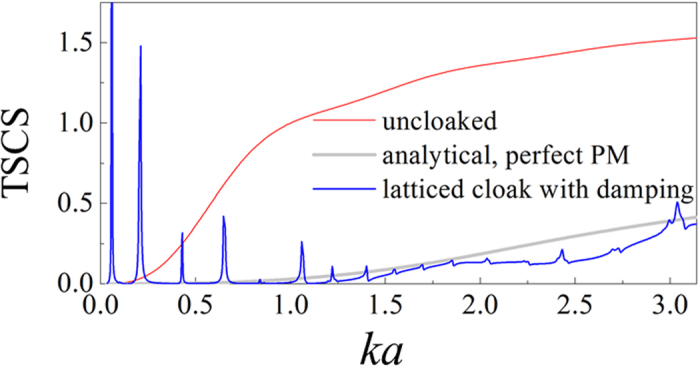
Improved broadband TSCS spectra for the latticed cloak. 0.5% imaginary part is introduced into the modulus of the base material.

A possible way to improve performance of the imperfect PM cloak is to damp the resonances. To give a preliminary verification, we introduce viscous damping to the base material of the latticed cloak by 0.5% imaginary modulus, i.e. (1 + 0.005*i*) *E*_*b*_ with *E*_*b*_ being the original Young’s modulus. The recalculated TSCS spectra are shown in Fig. 5. It is seen that except some ones at very low frequency, most resonance peaks are damped out, and the cloak works over a considerably wide frequency range. We would like to note that the current calculation is a conceptual demonstration since the damping introduced is greater than that in most metal materials and independent of frequency. Strategy such as combining viscoelastic polymers into cloak structure can be pursued in practical application.

In this article, we have reported detailed microstructure design of a latticed PM cloak, and its performance is numerically verified. The impact of resonances due to weak shear resistance inherent in PM lattices on cloaking performance is also examined. It is revealed that the broadband feature predicted based on an ideal PM cloak is punctured by the resonance peaks induced by weak shear resistance in PM lattice, and the cloak works in segmented frequency intervals. We have also demonstrated that the resonances can be suppressed by applying appropriate damping. We hope the present work makes a firm step towards experimental demonstration of PM cloaks and other acoustic devices making use of solid based PM materials as well.

## Additional Information

**How to cite this article**: Chen, Y. *et al.* Latticed pentamode acoustic cloak. *Sci. Rep.* **5**, 15745; doi: 10.1038/srep15745 (2015).

## Supplementary Material

Supplementary Information

## Figures and Tables

**Table 1:** Microstructure and homogenized property corresponding to each layer of the latticed cloak.

# (*r*/*a*)	*β*	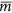	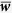	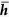	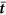	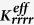	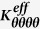	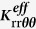	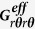
1(1.000)	1.3701	0.3500	0.8867	0.4028	0.0636	0.1168	8.5145	0.9402	0.0403
2(1.073)	1.3485	0.3641	0.8263	0.4478	0.0606	0.1380	7.2542	0.9660	0.0298
3(1.114)	1.2888	0.3781	0.7418	0.5268	0.0551	0.2008	5.0385	0.9902	0.0206
4(1.163)	1.2405	0.4041	0.7373	0.5541	0.0491	0.2668	3.7968	0.9971	0.0137
5(1.221)	1.1965	0.4192	0.6836	0.6008	0.0504	0.3342	3.0257	0.9975	0.0140
6(1.287)	1.1579	0.4324	0.7702	0.5561	0.0410	0.4010	2.5166	0.9994	0.0081
7(1.363)	1.1290	0.4594	0.7782	0.5549	0.0402	0.4659	2.1603	0.9983	0.0072
8(1.450)	1.1055	0.4888	0.7876	0.5510	0.0400	0.5277	1.9013	0.9967	0.0065
9(1.548)	1.0852	0.5100	0.7821	0.5542	0.0413	0.5860	1.7243	1.0000	0.0066
10(1.660)	1.0669	0.5320	0.8012	0.5436	0.0406	0.6398	1.5673	0.9961	0.0060
11(1.785)	1.0544	0.5594	0.8170	0.5345	0.0406	0.6886	1.4575	0.9963	0.0056
12(1.926)	1.0487	0.6027	0.8104	0.5367	0.0429	0.7326	1.3725	0.9965	0.0056

*The first column designates the starting location of each layer. *ρ*^eff^ is tuned to be 1.33 for each layer and not listed.
