# Supplementary material for: Latticed pentamode acoustic cloak
Source: Sci Rep. 2015 Oct 27;5:15745. doi: 10.1038/srep15745 (PMC4621533; doi:10.1038/srep15745)
Supplement: Supplementary Information [file srep15745-s1.pdf]

Latticed pentamode acoustic cloak (supplementary Info)

Yi Chen, Xiaoning Liu and Gengkai Hu

Key Laboratory of Dynamics and Control of Flight Vehicle, Ministry of Education, School of Aerospace Engineering, Beijing Institute of Technology, Beijing 100081, China

We present in this supplementary material analytical solutions of wave scattering of layered cylindrical cloak with perfect and imperfect PM materials. Consider elastodynamic equations for solid material

$$\boldsymbol{\sigma} = \mathbf{C} : \nabla \mathbf{u}, \qquad \rho \ddot{\mathbf{u}} = \nabla \cdot \boldsymbol{\sigma}. \tag{S1}$$

The elastic tensor of perfect PM material for cylindrical cloak is characterized by

$$\mathbf{C} = K_0 \mathbf{S} \otimes \mathbf{S}, \qquad \mathbf{S} = \sqrt{K_{rrrr}}\mathbf{e}_r \otimes \mathbf{e}_r + \sqrt{K_{\theta\theta\theta\theta}}\mathbf{e}_\theta \otimes \mathbf{e}_\theta, \tag{S2}$$

which has only one non-zero eigenvalue, and the material can sustain only one stress states proportional to $\mathbf{S}$, i.e. $\boldsymbol{\sigma} = -p\mathbf{S}$, where the scalar p is named as *pseudo pressure*. From Eq. (S1) and Eq. (S2), the wave equation of the perfect PM material can be expressed in the pseudo pressure as $\ddot{p} = \mathbf{S} : \nabla[\rho^{-1}\nabla \cdot (p\mathbf{S})]$. For 2D problem in polar system, it reads .

$$\frac{\partial^2 p}{\partial r^2} + \frac{1}{r}\frac{\partial p}{\partial r} - \left(\frac{c_r - c_\theta}{c_r}\right)^2 \frac{p}{r^2} + \left(\frac{c_\theta}{c_r}\right)^2 \frac{1}{r^2}\frac{\partial^2 p}{\partial \theta^2} = -\omega^2 \frac{p}{c_r^2}, \tag{S3}$$

where $c_r^2 = c_0^2 K_{rrrr}/\rho$ and $c_\theta^2 = c_0^2 K_{\theta\theta\theta\theta}/\rho$ are the phase velocities along the radial and ring directions, respectively, $c_0^2 = K_0/\rho_0$ the wave speed of background fluid. For imperfect PM material, the vectorial elastic wave which is governed by

$$\begin{pmatrix} \sigma_{rr} \\ \sigma_{\theta\theta} \\ \sigma_{r\theta} \end{pmatrix} = K_0 \begin{pmatrix} K_{rrrr} & K_{rr\theta\theta} & 0 \\ K_{rr\theta\theta} & K_{\theta\theta\theta\theta} & 0 \\ 0 & 0 & G_{r\theta r\theta} \end{pmatrix} \begin{pmatrix} \varepsilon_{rr} \\ \varepsilon_{\theta\theta} \\ \gamma_{r\theta} \end{pmatrix}$$

$$\varepsilon_{rr} = \frac{\partial u_r}{\partial r}, \qquad \varepsilon_{\theta\theta} = \frac{1}{r}\frac{\partial u_\theta}{\partial \theta} + \frac{u_r}{r}, \qquad \gamma_{r\theta} = \frac{\partial u_\theta}{\partial r} + \frac{1}{r}\frac{\partial u_r}{\partial \theta} - \frac{u_\theta}{r} \tag{S4}$$

$$\frac{\partial \sigma_r}{\partial r} + \frac{1}{r}\frac{\partial \tau_{r\theta}}{\partial \theta} + \frac{\sigma_r - \sigma_\theta}{r} = -\rho\omega^2 u_r, \qquad \frac{\partial \tau_{r\theta}}{\partial r} + \frac{1}{r}\frac{\partial \sigma_\theta}{\partial \theta} + \frac{2\tau_{r\theta}}{r} = -\rho\omega^2 u_\theta$$

must be considered.

S.1 Scattering of cylindrical layered cloak with perfect PM materials

Suppose the cloak is assembled from N perfect PM layers which are numbered from the inner side to the outer by $j=1\sim N$. The $j^{\text{th}}(j=1\sim N)$ PM layer occupies ring region $r_{j-1}<r<r_j$ and is characterized by ($K_{jrrrr}, K_{j\theta\theta\theta\theta}, \rho_j$). An incident plane wave $p_{in}=\exp(ik_0x)$ with $\exp(-i\omega t)$ time dependence is excited in the background fluid, where $k_0=\omega/c_0$ is the wave number. The incident and scattered pressure in background region ($r>r_N$) are expressed in Bessel function and Hankel function of the first kind, respectively,

$$p_{in}=\sum_{n=0}^{\infty}a_nJ_n(k_0r)\cos n\theta,\quad p_{sc}=\sum_{n=0}^{\infty}b_nH_n^{(1)}(k_0r)\cos n\theta \tag{S5}$$

with $a_n=(2-\delta_{0n})i^n(n>0)$ being the known incident coefficient.

The pseudo pressure in each PM layer satisfies equation Eq. (S3) in polar system. The solution can be expanded as summation of $g_{jn}(r)\cos n\theta$, and $g_{jn}(r)$ satisfies Bessel equation of non-integer order v_{jn}

$$g''_{jn}+\frac{1}{r}g'_{jn}+\left(k_{jr}^2-\frac{v_{jn}^2}{r^2}\right)g_{jn}=0,\quad v_{jn}^2=n^2\left(\frac{c_{j\theta}}{c_{jr}}\right)^2+\left(\frac{c_{jr}-c_{j\theta}}{c_{jr}}\right)^2$$

where $c_{jr}^2=c_0^2K_{jrrrr}/\rho_j$, $c_{j\theta}^2=c_0^2K_{j\theta\theta\theta\theta}/\rho_j$ and $k_{jr}=\omega/c_{jr}$. The pseudo pressure field in the jth PM layer is then obtained as,

$$p_j(r,\theta)=\sum_{n=0}^{\infty}\left[a_{jn}J_{jv}(k_{jr}r)+b_{jn}H_{jv}^{(1)}(k_{jr}r)\right]\cos n\theta \tag{S6}$$

where J_{jv} and $H_{jv}^{(1)}$ designate Bessel function and Hankel function of non-integer order v_{jn} for the jth layer. Employing the continuity condition of normal velocity and traction across the interfaces between PM layers (for background fluids, $\mathbf{S}=\mathbf{I}$),

$$[\rho^{-1}\mathbf{e}_r\cdot\mathbf{S}\cdot\nabla p]_{r=r_j}=0,\qquad [p\mathbf{e}_r\cdot\mathbf{S}]_{r=r_j}=0,\quad (j=1\sim N).$$

the transmittance relation for the coefficients of adjacent layers can be obtained as,

$$\begin{pmatrix}a_{jn}\\ b_{jn}\end{pmatrix}=\mathbf{Y}_{jn}\begin{pmatrix}a_{(j+1)n}\\ b_{(j+1)n}\end{pmatrix},\quad \mathbf{Y}_{jn}=A_{jn}\begin{pmatrix}\tau_{j11} & \tau_{j12}\\ \tau_{j21} & \tau_{j22}\end{pmatrix},\quad (j=1,2...N) \tag{S7}$$

$$\tau_{j11}=\rho_{j+1}s_{(j+1)r}J_{(j+1)v}(k_{(j+1)r}r_j)h_j(r_j)-\rho_js_{j,r}H_{jv}^{(1)}(k_{jr}r_j)f_{j+1}(r_j)$$
$$\tau_{j22}=\rho_js_{jr}J_{jv}(k_{jr}r_j)h_{j+1}(r_j)-\rho_{j+1}s_{(j+1)r}H_{(j+1)v}^{(1)}(k_{(j+1)v}r_j)f_i(r_j)$$
$$\tau_{j12}=\rho_{j+1}s_{(j+1)r}H_{(j+1)v}^{(1)}(k_{(j+1)r}r_j)h_j(r_j)-\rho_js_{jr}H_{jv}^{(1)}(k_{jr}r_j)h_{j+1}(r_j)$$
$$\tau_{j21}=\rho_js_{jr}J_{jv}(k_{jr}r_j)f_{j+1}(r_j)-\rho_{j+1}s_{(j+1)r}J_{(j+1)v}(k_{(j+1)r}r_j)f_j(r_j)$$

$$A_{jn}^{-1} = r_j \rho_{j+1} s_{j,r}^2 (J_{jv}(k_{jr}r) H_{jv}'^{(1)}(k_{jr}r_j) - H_{jv}^{(1)}(k_{jr}r_j) J_{jv}'(k_{jr}r_j))$$
$$h_j(r) = (s_{jr} - s_{j\theta}) H_{jv}(k_{jr}r) + s_{jr} r H_{jv}'^{(1)}(k_{jr}r)$$
$$f_j(r) = (s_{jr} - s_{j\theta}) J_{jv}(k_{jr}r) + s_{jr} r J_{jv}'(k_{jr}r)$$

where $s_{jr} = \sqrt{K_{jrrrr}}$, $s_{j\theta} = \sqrt{K_{j\theta\theta\theta}}$ and the prime denotes derivative with respect to r. The solution of scattering coefficients b_n in the background fluids can be finalized by complementing the sound-hard condition (radially fix the displacement) at the cloak's inner boundary, $(\rho^{-1}\mathbf{e}_r \cdot \mathbf{S} \cdot \nabla p)_{|r=r_0} = 0$,

$$b_n = -a_n \frac{T_{n11}\xi_{an} + T_{n21}\xi_{bn}}{T_{n12}\xi_{an} + T_{n22}\xi_{bn}}, \qquad \mathbf{T}_n = \prod_{j=1}^{N} \mathbf{Y}_{jn} \tag{S8}$$

$$\xi_{an} = r_0^{-1}(s_{1r} - s_{1\theta}) J_{1v}(k_{1r}r_0) + s_{1r} J_{1v}'(k_{1r}r_0)$$
$$\xi_{bn} = r_0^{-1}(s_{1r} - s_{1\theta}) H_{1v}^{(1)}(k_{1r}r_0) + s_{1r} H_{1v}'^{(1)}(k_{1r}r_0)$$

The pseudo pressure in each layer can then be determined by Eqs. (S6-S8).

S.2 Scattering of cylindrical layered cloak with imperfect PM materials

For the cylindrical cloak with imperfect PM material, the pressure in background fluid still takes Eq. (S5), while the wave field inside the imperfect PM material obeys elastic wave Eq. (S4). The problem is actually to solve acoustic scattering of a cylindrical shell made of graded orthotropic solids, and is hardly to get a closed form analytical solution. To this end, a semi-analytical procedure, state space approach [1, 2], is employed.

Corresponding to cylindrical expansion of the incident wave, we adopt following mode expansions of displacement and stress inside the imperfect PM cloak shell

$$\begin{gathered} u_r = \sum_{n=0}^{\infty} u_{nr}(r)\cos n\theta, \quad u_\theta = \sum_{n=0}^{\infty} u_{n\theta}(r)\sin n\theta \\ \sigma_r = \sum_{n=0}^{\infty} \sigma_{nr}(r)\cos n\theta, \ \sigma_{r\theta} = \sum_{n=0}^{\infty} \sigma_{nr\theta}(r)\sin n\theta, \ \sigma_\theta = \sum_{n=0}^{\infty} \sigma_{n\theta}(r)\cos n\theta \end{gathered} \tag{S9}$$

For the nth order, $\mathbf{D}_n = \{u_{nr}, u_{n\theta}, \sigma_{nr}, \sigma_{nr\theta}\}^{\mathrm{T}}$ is defined as the state variable since they are connected layer by layer by the continuous condition across interfaces. By using Eq. (S9) and Eq. (S4), and after a tedious manipulation, $\mathbf{D}_n$ is derived to be governed by a set of ordinary differential equation,

$$\frac{\mathrm{d}\mathbf{D}_n}{\mathrm{d}r} = \mathbf{P}_n(r)\mathbf{D}_n \tag{S10}$$

$$\mathbf{P}_n\left(r\right)=\begin{pmatrix} -\frac{1}{r}\eta & -\frac{n}{r}\eta & \frac{1}{K_0K_{rrrr}} & 0 \\ \frac{n}{r} & \frac{1}{r} & 0 & \frac{1}{K_0G_{r\theta r\theta}} \\ \frac{1}{r^2}\xi-\rho\omega^2 & \frac{n}{r^2}\xi & \frac{1}{r}(\eta-1) & -\frac{n}{r} \\ \frac{n}{r^2}\xi & \frac{n}{r^2}\xi-\rho\omega^2 & \frac{n}{r}\eta & -\frac{2}{r} \end{pmatrix}$$

where $\xi=K_0(K_{rrrr}K_{\theta\theta\theta\theta}-K_{rr\theta\theta}^2)/K_{rrrr}$ and $\eta=K_{rr\theta\theta}/K_{rrrr}$. Eq. (S10) can be numerically solved by discretizing the cloak (either continuously varying or layered) shell further into N sufficiently thin layers, hence $\mathbf{P}_n\left(r\right)$ can be regarded as a constant matrix in each thin layer. The state variable at the front ($r=r_j$) and back ($r=r_{j-1}$) of the jth thin layer is connected by

$$\mathbf{D}_n(r_j)=\exp\left[(r_j-r_{j-1})\mathbf{P}_{\text{n}}\left(\frac{r_j+r_{j-1}}{2}\right)\right]\mathbf{D}_n(r_{j-1}), \tag{S11}$$

Accumulatively, $\mathbf{D}_n$ at the outer and inner side of the cloak is connected as

$$\mathbf{D}_n(r_N)=\mathbf{T}_n\mathbf{D}_n(r_0), \qquad \mathbf{T}_n=\prod_{j=1}^{j=N}\exp((r_j-r_{j-1})\mathbf{P}_{\text{n}}((r_j+r_{j-1})/2)). \tag{S12}$$

At the outer boundary of the cloak, the continuous condition between the solid and background fluid is fulfilled by

$$\begin{aligned} &u_{nr}(r_N)=(a_nJ_n'(k_0r_N)+b_nH_n'^{(1)}(k_0r_N))/\rho_0\omega^2 \\ &\sigma_{nr}(r_N)=-(a_nJ_n(k_0r_N)+b_nH_n^{(1)}(k_0r_N)), \quad \sigma_{nr\theta}(r_N)=0 \end{aligned}.$$

At the inner boundary of the cloak, radially supported boundary condition is enforced in consistent with that for the cloak with perfect PM material,

$$u_{nr}(r_0)=0, \quad \sigma_{nr\theta}(r_0)=0.$$

The scattering coefficient in the background fluid can then be determined as,

$$b_n=-a_n\frac{(T_{n33}T_{n42}-T_{n32}T_{n43})J_n'(k_0r_N)-(T_{n12}T_{n43}-T_{n13}T_{n42})K_0k_0^2J_n(k_0r_N)}{(T_{n33}T_{n42}-T_{n32}T_{n43})H_n'^{(1)}(k_0r_N)-(T_{n12}T_{n43}-T_{n13}T_{n42})K_0k_0^2H_n^{(1)}(k_0r_N)} \tag{S13}$$

In the numerical calculation, the cloak shell is discretized into about 120 thin layers.

S.3 Determination of TSCS

Having the scattering coefficient b_n, the total scattered energy can be calculated using following integral along a circle with radius r_{sc} enclosing the cloak shell,

$$E_{sc} = -\frac{1}{2\rho\omega}\mathrm{Im}\oint_C p_{sc}\frac{\partial \overline{p}_{sc}}{\partial n}ds = -\frac{r_{sc}}{2\rho\omega}\mathrm{Im}\int_0^{2\pi} p_{sc}\overline{p}'_{sc}d\theta . \quad \text{(S14)}$$

Substituting Eq. $(S5)_2$ into above equation, and considering the identity

$$\mathrm{Im}\left[H_n^{(1)}(k_0 r_{sc})\overline{H_n'^{(1)}(k_0 r_{sc})}\right] = -2/(\pi r_{sc}),$$

Eq. (S14) can be proved to be independent on r_{sc}. The total scattered energy and TSCS are evaluated as

$$E_{sc} = \frac{1}{\rho\omega}\sum_{n=0}^{\infty}(1+\delta_{0n})|b_n|^2, \qquad \mathrm{TSCS} = \frac{1}{k_0 r_0}\sum_{n=0}^{\infty}(1+\delta_{0n})|b_n|^2 \quad \text{(S15)}$$

For the full wave simulation of the latticed cloak, Eq. (S14) is directly used in the TSCS calculation by numerical integration.

S.4 Effective density of the PM material

Since the PM lattice is solid and there is no resonance happens in the considered frequency regime, the effective density can be simply calculated by its volume average. As a justification, we plot in the figures below the dynamic effective densities of the PM material along the *r*- and *theta*- direction within *ka*=[0, Pi]. We adopt the same method retrieving the dynamic effective density as that in [3]. The left panel and right panel are the densities retrieved from the most inner layer and most outer layer of the cloak, respectively. It is seen from the figure that the density anisotropy of the PM material is negligible and very close to the volume average.

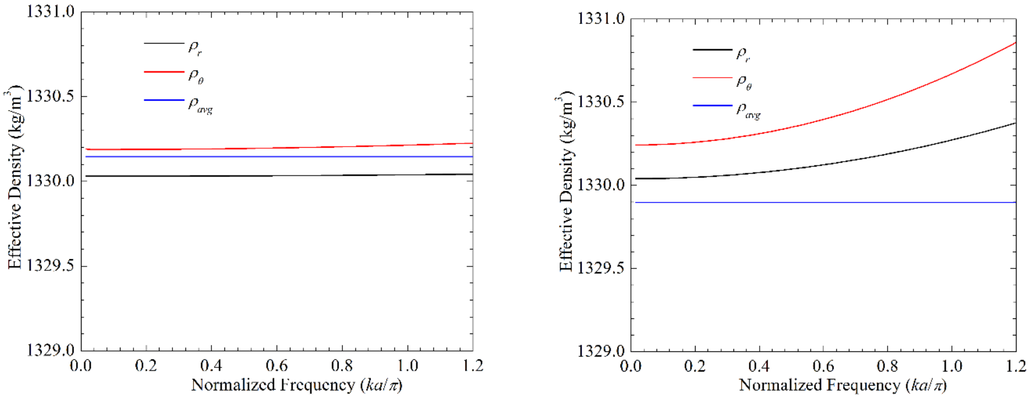

References:

[1] W. Chen, Z. Bian, and H. Ding, Int. J. Mech. Sci. 46, 159 (2004).

[2] S. Hasheminejad and M. Rajabi, J. Sound Vib. 302, 208 (2007).

[3] R. Zhu, X. Liu, G. Huang, H. Huang and C. Sun, Phys. Rev. B, 86, 144307(2012).
